# Pediatric endoscopic pilonidal sinus treatment (PEPSiT): report of a multicentric national study on 294 patients

**DOI:** 10.1007/s13304-023-01508-5

**Published:** 2023-05-05

**Authors:** Ciro Esposito, Ernesto Leva, Piergiorgio Gamba, Alberto Sgrò, Umberto Ferrentino, Alfonso Papparella, Fabio Chiarenza, Cosimo Bleve, Mario Mendoza-Sagaon, Ernesto Montaruli, Maria Escolino

**Affiliations:** 1 Division of Pediatric Surgery, Federico II University Hospital, Via Pansini 5, 80131 Naples, Italy; 2 Division of Pediatric Surgery, Fondazione IRCCS Ca’ Granda Ospedale Maggiore Policlinico, Milan, Italy; 3 Division of Pediatric Surgery, Medical University of Padua, Padua, Italy; 4 Division of Pediatric Surgery, University Hospital San Giovanni di Dio e Ruggi d’Aragona, Salerno, Italy; 5 Division of Pediatric Surgery, University Hospital Luigi Vanvitelli, Naples, Italy; 6 Division of Pediatric Surgery, San Bortolo Hospital, Vicenza, Italy; 7 Division of Pediatric Surgery, Ospedale Regionale di Bellinzona e Valli, Bellinzona, Switzerland

**Keywords:** Pilonidal sinus disease, PEPSiT, Laser, Success, Recurrence, Dressing

## Abstract

This study aimed to report a multicentric national experience about the outcomes of pediatric endoscopic pilonidal sinus treatment (PEPSiT). The medical records of all pediatric patients, aged up to 18 years, who underwent PEPSiT in the period 2019–2021, were retrospectively reviewed. Patients’ demographics, operative details, and post-operative outcomes were assessed. A total of 294 patients (182 boys), with median age of 14 years (range 10–18), receiving PEPSiT in the study period, were enrolled. Pilonidal sinus disease (PSD) was primary in 258 (87.8%) and recurrent in 36 (12.2%). The median operative time was 36 min (range 11–120). The median VAS pain score was 0.86 (range 0–3) and the median duration of analgesic use was 27 h (range 12–60). The overall success rate was 95.2% (280/294) and the median time to full healing was 23.4 days (range 19–50). Six/294 (2.0%) patients developed Clavien 2 post-operative complications. The recurrence rate was 4.8% (14/294) and all recurrences were re-operated using PEPSiT. Redo-surgery for wound debridement was performed in one (0.3%) patient with late healing. On multivariate analysis, hirsutism and typology of sinus (pits ≥ 2, paramedian and more proximal to the anus) were predictors of PSD recurrence (*p* = 0.001). To date, this is the largest series of PEPSiT published in the pediatric population. The outcomes reported after a 3 years experience confirm that PEPSiT is a safe, effective, and real minimally invasive procedure to treat adolescents with PSD. It provides patients quick and painless recovery, satisfactory success, and high quality of life.

**Graphical abstract:**

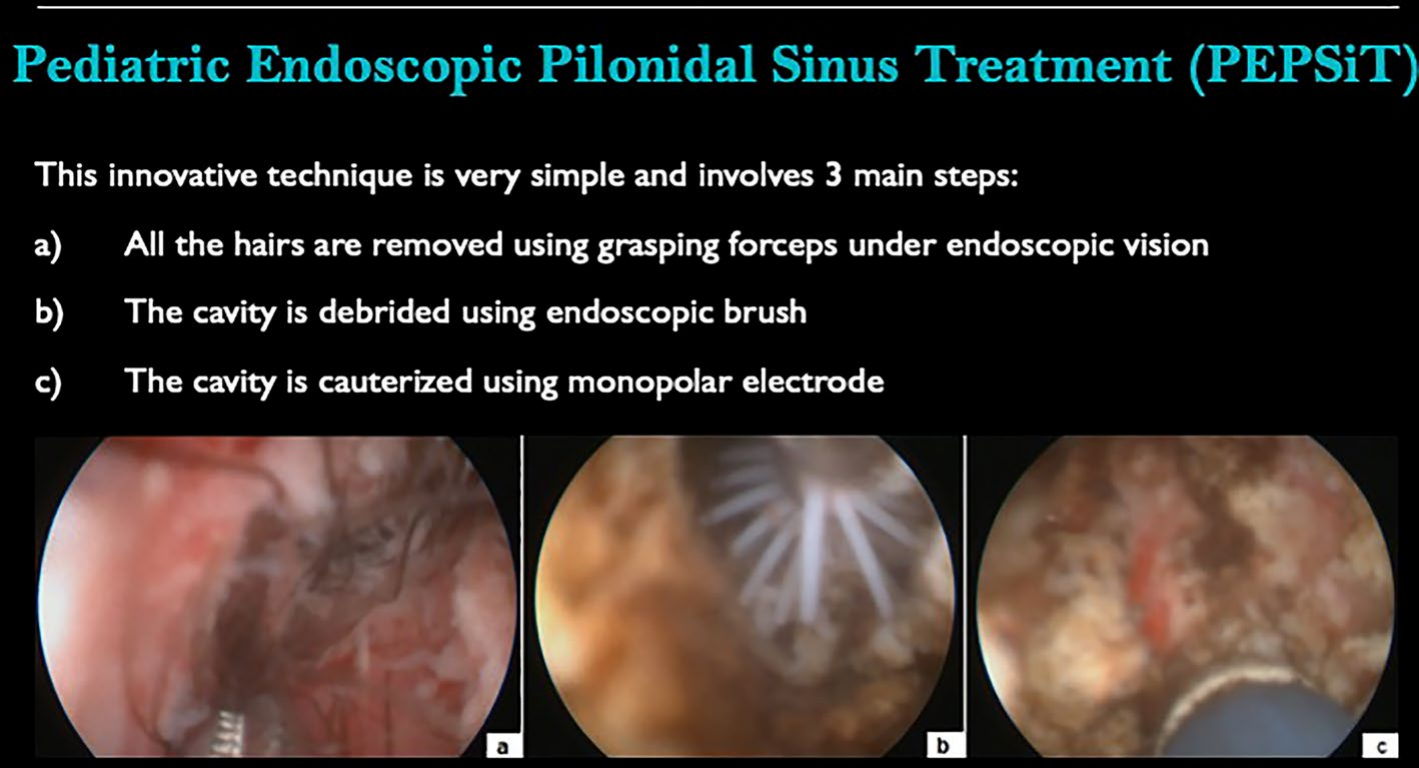

## Introduction

Pilonidal sinus disease (PSD) is a relatively frequent pathology that occurs primarily in young adults with a 3:1 male predominance [1, 2]. When complicated with acute or chronic infection, it requires surgical treatment. Several techniques for surgical treatment of PSD have been reported in the past years, but none has been established as the gold standard [3, 4]. The reasons are mainly the high rates of post-operative complications (14–30%) and disease recurrence (15–40%) reported with these approaches [5, 6]. Classical surgical methods often require long hospital stay, are painful, and have a significant negative impact on the patient’s quality of life for a long time post-operatively [7].

Thus, the best approach would be an operative technique that is easy to apply and allows patients to return quickly to their daily activities. In 2013, Meinero introduced a new video-assisted minimally invasive technique for the PSD treatment (EPSIT) with very promising results [8]. Following this report, in 2017, we decided to apply the same technique in the pediatric population, calling it pediatric endoscopic pilonidal sinus treatment (PEPSiT) [9]. We demonstrated that this new method is ideal in terms of technical ease, short recovery, and minimal patient discomfort and pain, with good long-term results in both primary and recurrent PSD [10, 11]. We also described a treatment protocol for PSD, including pre- and post-operative laser epilation, standardized operative technique and specific wound management [12, 13].

Since its first report in the pediatric population in 2017, PEPSiT has been increasingly adopted in the pediatric population [14–16]. However, most papers available in the pediatric literature reported initial experience with low number series and short follow-up period [17–19]. The efficacy of this technique needs to be confirmed by larger number series and longer follow-up.

This study aimed to collect and report a multicentric national experience about the outcomes of PEPSiT over the last 3 years.

## Patients and methods

The medical records of all pediatric patients, aged up to 18 years, who underwent PEPSiT for treatment of PSD over a 3 year period (January 2019–December 2021) in 7 pediatric surgery units, were retrospectively reviewed. The cases were evaluated regarding patient demographics, duration of the procedure, post-operative pain on the Visual Analogue Scale (VAS), duration of analgesic use, hospital stay, return to normal daily activity, time to full healing, post-operative complications such as bleeding, infection, dehiscence, granuloma, disease recurrence and re-operations.

The primary outcome of this study was to assess the success rate of PEPSiT and time to full healing. The surgical success was defined as complete healing by 8 post-operative weeks. Healing was defined as the complete skin closure of the surgical wound, without signs of inflammation, secretion, and pain, both subjectively and during clinical examination by a physician.

The secondary outcome was to evaluate risk of complications, post-operative pain on the VAS scale, hospital stay, disease recurrence and re-operations. Recurrence was defined as lasting or recurrent secretion more than 3 months after surgery with infection or abscess formation and local pain and/or discomfort.

Multivariate logistic regression analysis was performed to assess risk factors for PSD recurrence. Factors assessed included gender, obesity, hirsutism, typology of sinus (number of pits, distance of the pits from the midline).

Statistical analysis was carried out using the computer software SPSS 17.0 for Windows XP. Significance was defined as *p* value < 0.05.

The appropriate Institutional Review Board (IRB) approval at each participating center was obtained for this study.

### Pre-operative management

Active infections or abscesses were treated with antibiotics before proceeding with surgery. Additionally, all patients received at least 2 pre-operative laser epilation therapy sessions at 4–6 weeks intervals. A Multi Variant Pulsed Light (MVPL) Laser was adopted in all cases. The treatment area included the intergluteal crease and a 5 cm area from the midline on both sides (left/right) of the crease by 12 cm (cranial/caudal) from the apex to the anal opening. The patients were also requested to do mechanical epilation of the intergluteal area by shaving every week at home.

### Operative procedure

Patients were placed in a prone position with buttocks retracted with adhesive tape and pre-operative antibiotic prophylaxis was administered. The fistuloscopy set (KARL STORZ SE & Co. KG, Tuttlingen, Germany) included a 10 Fr fistuloscope, with 8° angled eyepiece and a working length of 18 cm, an endoscopic grasping forceps, a 7 Fr monopolar electrode, and a fistula brush with single use insert of different outer diameter (4, 4.5 and 5 mm).

The fistula’s external pit was enlarged using a curved spreading clamp and the fistulous tract was dilated by injecting saline with a syringe. In case of multiple pits, the lower one was adopted to introduce the fistuloscope. Once started the fistuloscopy, the main cavity was explored and so as all potential secondary tracts or abscess cavities. A constant flow of 0.54% mannitol-2.7% sorbitol irrigation solution aided to prevent the collapse of the cavity. All present hairs were removed using the grasping forceps under endoscopic vision. Then, the cavity was debrided using endoscopic brusher for mechanical abrasion purposes to remove necrotic tissues and fibrin. Finally, the cauterization of the main cavity and all the secondary tracts was carried out using the monopolar electrode. Accurate hemostasis was finally provided. It was crucial during the procedure turn the fistuloscope upside down to check and treat the upper side of the cavity. At the end of the fistuloscopy, the external pit was coagulated using standard electrocautery at spray modality setting and a compressive dressing was performed.

### Post-operative management

**Fig. 1:**
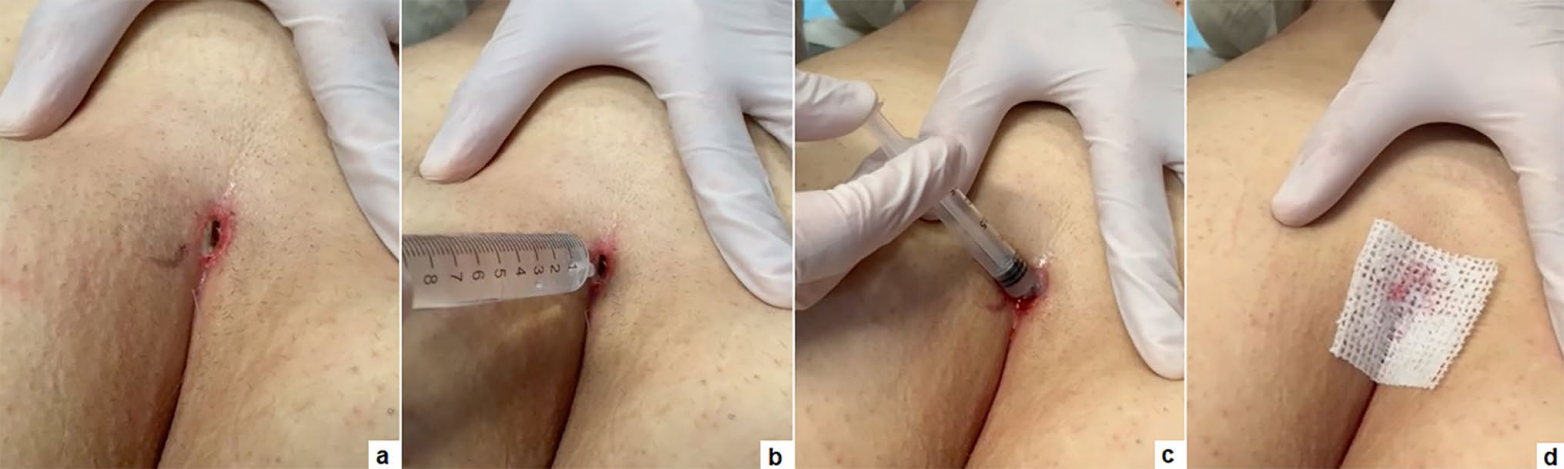
Dressing after PEPSiT: the buttocks are slightly enlarged **a** 10–20 mL of saline are injected through the external pit to wash the cavity **b**; a little amount (< 0.5 cc) of ozonated oil is injected into the cavity using a syringe **c** the wound is covered with a wet gauze **d**

Before hospital discharge, patients’ relatives/caregivers were instructed on how to take care of the surgical wound by showing them the dressing performed in the hospital or providing them a video recording of the dressing. They performed irrigation of the cavity with 10–20 mL of saline and injected a little amount (< 0.5 cc) of ozonated oil by introducing the tip of the syringe through the external pit. Finally, the wound was covered with a hyaluronic acid-based wet gauze (Fig. 1). This dressing was repeated twice a day at home. Mechanical epilation was advised with regular shaving of the intergluteal space until the external opening healing was complete. Follow-up clinical evaluations were performed at 1 week, 2, 4, 6, 8 weeks and tri-monthly for 2 years post-operatively.

When the wound was healed, all patients received laser epilation therapy with sessions repeated at 4–6 weeks intervals. Laser therapy was stopped when there was no longer any visible hair within the treatment area around the initial PSD site. Furthermore, we advised the patients to keep care to the local hygiene, to wash the perineal region after each visit to the toilet and to accurately dry it after washing.

## Results

A total of 294 patients (182 boys and 112 girls) received PEPSiT in the study period and were enrolled in the study. The median patient age at the time of surgery was 14 years (range 10–18) and the median weight was 75.6 kg (range 44–98). Two-hundreds and 58 (87.8%) patients had primary pilonidal sinus disease (PSD) whereas 36 (12.2%) patients had recurrent PSD following primary surgery at a different institution.

The procedure was performed using saddle spinal block or locoregional anesthesia in most patients (270/294, 91.8%) and the median operative time was 36 min (range 11–120). The median VAS pain score was 0.86 (range 0–3) and the median duration of analgesic use (paracetamol, ibuprofen) was 27 h (range 12–60). All patients were mobilized on the same day of surgery. PEPSiT was performed as outpatient or with an overnight hospitalization in all centers. The median hospital stay length was 15 h (range 13–48).

**Fig. 2:**
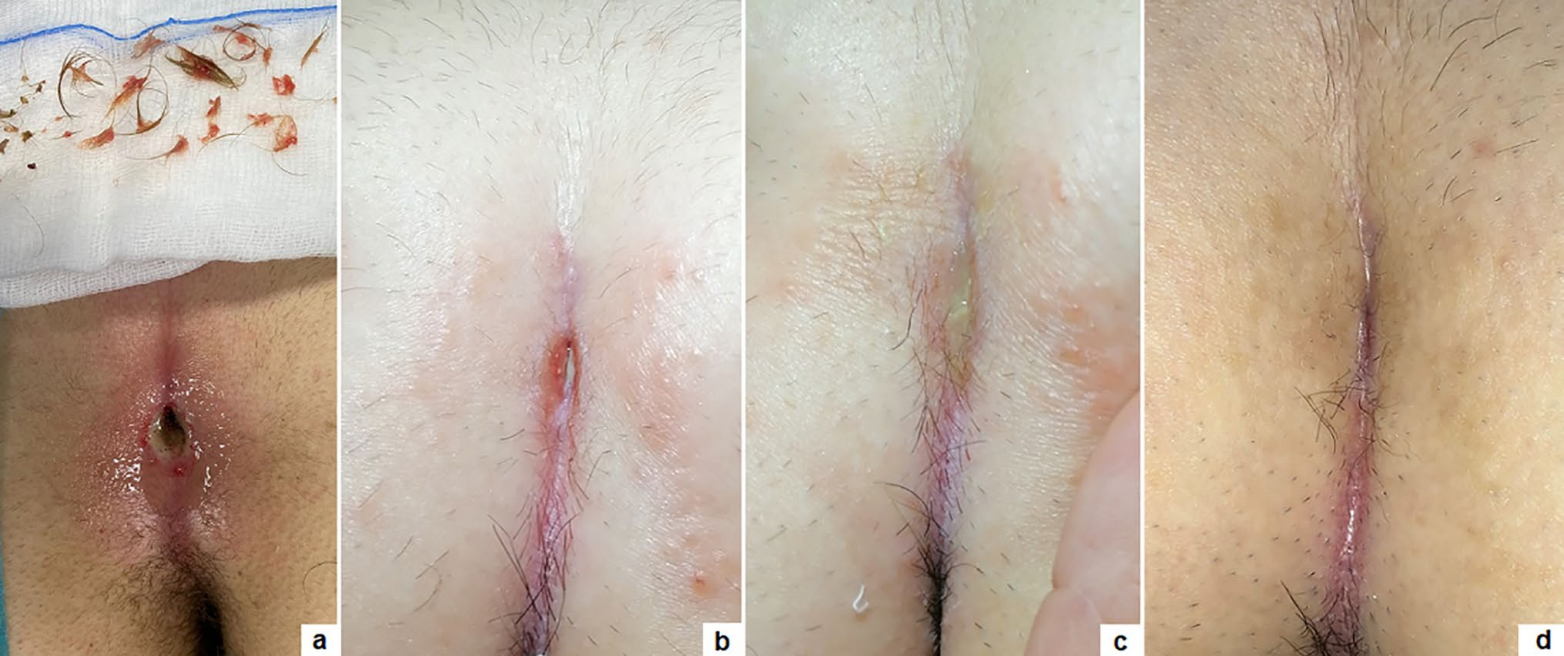
Wound healing: at time of surgery **a**; 7th **b**; 14th **c** and 21st post-operative day **d**

The median follow-up duration was 17.7 months (range 1–36). The overall success rate was 95.2% (280/294). The median post-operative time to full healing was 23.4 days (range 19–50) (Fig. 2). Early wound healing (average 17 days) was observed in 290 (98.6%) patients while late wound healing (average 60 days) was seen in 4 (1.4%) patients. The median time of return to full daily activities was 1.5 days (range 0–8) while it was the same day in 160 (54.4%) patients.

Six/294 (2.0%) patients developed Clavien 2 post-operative complications, including wound infection (*n* = 3), granuloma (*n* = 1), persistent subcutaneous edema (*n* = 1) and local orifice burn (*n* = 1). PSD recurrence rate was 4.8% (14/294) and all recurrences were re-operated using PEPSiT with no further recurrence. One (0.3%) patient presenting late healing needed re-operation for wound debridement. Patients’ demographics and results are reported in Table 1.

**Table 1:** Patients’ demographics and results

Parameter	
Total number, *n*	294
Male/Female, *n*/*n*	182/112
Age, years	14 (10–18)
Median (IQR)	
Weight, kg	75.6 (44–98)
Median (IQR)	
Primary PSD, *n* (%)	258 (87.8)
Recurrent PSD, *n* (%)	36 (12.2)
Operative time, min	36 (11–120)
Median (IQR)	
VAS pain score, *n*	0.86 (0–3)
Median (IQR)	
Duration of analgesic use, hours	27 (12–60)
Median (IQR)	
Hospital stay length, hours	15 (13–48)
Median (IQR)	
Follow-up duration, months	17.7 (1–36)
Median (IQR)	
Overall surgical success, *n* (%)	280 (95.2)
Time to full healing, days	23.4 (19–50)
Median (IQR)	
Time of return to full daily activities, days	1.5 (0–8)
Median (IQR)	
Post-operative complications, overall *n* (%)	6 (2.0) Clavien 2
Wound infection, *n* (%)	3 (1.0)
Granuloma, *n* (%)	1 (0.3)
Persistent subcutaneous edema, *n* (%)	1 (0.3)
Local orifice burn, *n* (%)	1 (0.3)
PSD recurrence, *n* (%)	14 (4.8)
Re-operations, *n* (%)	15 (5.1)

*IQR* interquartile range, *PSD* pilonidal sinus disease, *VAS* visual analog scale

On multivariate analysis, hirsutism and typology of sinus (≥ 2 external pits, paramedian pits and pits more proximal to the anus) were associated with higher rates of post-operative PSD recurrence (*p* = 0.001) (Table 2).

**Table 2:** Multivariate logistic regression analysis of risk factors for PSD recurrence in our series

Variable	OR	95% CI	*p* value
Male gender	0.935	0.547–1.398	0.856
Female gender	0.899	0.520–0.998	0.899
BMI ≥ 30 kg/m^2^	0.934	0.557–1.166	0.795
Hirsutism	1.128	1.008–1.221	0.001
External pit = 1	0.623	0.521–0.955	0.855
External pits ≥ 2	1.015	1.005–1.036	0.001
Median pit(s)	0.755	0.456–1.285	0.875
Paramedian pit(s)	1.038	1.001–1.399	0.001
Pit(s) proximal to the anus	1.215	1.015–1.450	0.001

*BMI* Body Mass Index

## Discussion

In the recent years, there has been a general trend toward minimal invasiveness in all surgical fields, and this was also the case of pilonidal sinus disease (PSD). Endoscopic pilonidal sinus treatment or EPSiT was first described in adults by Meinero in 2014 [8]. The same technique was applied in the pediatric population with very promising results and called PEPSiT (pediatric endoscopic pilonidal sinus treatment) by Esposito et al. [9]. Since this first report, PEPSiT has been increasingly adopted in the pediatric population because it is easy to apply, painless and with a very short hospital stay [14–16].

Some studies have reported multiple advantages of the endoscopic technique as compared to open surgery techniques [8, 14, 16]. The main advantage of PEPSiT over standard surgical approach is endoscopic access to the pilonidal cavity, allowing avoidance of long surgical incisions, direct vision of the main fistula tract and all accessory tracts and removal of debris and hair under direct vision. This is considered fundamental for quicker recovery and healing.

However, most of the papers published in the pediatric literature were single-center or multicentric series reporting initial experience with small sample size and short follow-up period [15–20]. Further studies with larger series and longer follow-up are needed to confirm the efficacy of the technique and to establish it as the new gold standard for treatment of PSD. For this reason, we decided to collect and report a multicentric national experience about the outcomes of PEPSiT over the last 3 years.

This study confirmed that PEPSiT was associated with very low pain levels, without the need for long and painful wound care. The cosmetic result was excellent, as well as patient satisfaction, with no impact on quality of life since most patients resumed their full daily activity the same day of surgery. In addition, this procedure requires no hospital stay, and it can be performed as outpatient.

The surgical technique is relatively easy to perform, with a quick learning curve. But, based on our collected experience, it is crucial to standardize the technique, use adequate instrumentation and set properly the operating room. We advise use of 0.54% mannitol-2.7% sorbitol solution as irrigation solution to avoid the risk of electric injury due to monopolar energy. To reduce the risk of subcutaneous edema, it is important to adopt a compressive dressing on the buttocks for at least 8–12 h post-operatively. During the procedure, it is important to visualize all fistulous tracts and secondary cavities, remove all the hairs and hair follicles, cauterize the whole tissue, and finally take out all the necrotic remnants and fibrin debris. To correctly accomplish these tasks, it is crucial to adopt some technical tricks such as to turn upside down the fistuloscope to check the roof, remove all hairs, brush, and cauterize the upper side of the fistula. It is also important to keep care to the skin during use of electrocautery to avoid thermal injuries, as reported in one case of our series.

Another advantage of PEPSiT is its easy applicability even for recurrent PSD following the failure of either open surgery or PEPSiT. Redo-PEPSiT was applied to successfully treat all 14 (4.8%) patients of our series with recurrent disease after the PEPSiT procedure.

As previously published [13], it is equally crucial to standardize wound dressing and adequately instruct family members on how to perform it. Before hospital discharge, we showed them the dressing in the hospital or provided them a video recording. The dressing is extremely easy and no time spending and requires irrigation of the cavity with 10–20 mL of saline and application of ozonated oil introducing the tip of the syringe through the external pit. According to our protocol, the dressing should be repeated twice a day until completion of the healing process.

Based on PSD etiopathogenesis [21, 22], hair follicle eradication using laser energy can be a key factor to prevent recurrences [13, 23]. Therefore, laser epilation was performed in all our patients both pre- and post-operatively until there was no longer any visible hair in the intergluteal crease. This treatment was well tolerated by all patients, who reported no pain or discomfort and no related complications such as thermal injuries or skin pigmentation changes. In a recent study by Gökbuget et al. [17], the authors reported a 27.5% recurrence rate that is higher than the average reported in the literature. The authors explained such a high recurrence rate as probably related to the lack of integration of laser epilation in their treatment protocol.

Another key point is to “stress” the patients and their parents on the importance to keep an accurate local hygiene and respect all follow-up appointments to maintain a successful outcome.

Limitations of the present study include the retrospective design and the multi-institutional participation with their inherent bias. Another limitation to be addressed is the limited follow-up period of our series. The current literature has reported that the long-term recurrence rate of minimally invasive techniques for treatment of PSD is still unclear [24]. The litmus test of every operative technique is the long-term follow-up, reporting low recurrence rate, to be adopted in each hospital worldwide [24]. Therefore, a recent meta-analysis suggested that an adequate long-term follow-up for PSD surgery should be at least 5 years [25]. Further studies with at least 5 years follow-up are needed to confirm the preliminary results of this series.

Another bias could be the application of the endoscopic technique to certain typology of PSD. In this setting, a recent consensus statement has been developed for the adults with PSD [26]. In our treatment protocol for PSD, the application of PEPSiT technique is in-line with the consensus’ recommendation to adopt endoscopic treatment in cases of limited PSD (single/multiple pits on the midline). Recurrent PSD can be managed with PEPSiT as well as de novo presentation disease [26]. Our study demonstrated that hirsutism and typology of sinus (≥ 2 external pits, paramedian pits and pits more proximal to the anus) were the main predictors of post-operative PSD recurrence. Thus, in accordance with the cited consensus statement [26], we strongly affirm that proper surgical treatment should be tailored to the individual patient.

To date, this is the largest series of PEPSiT published in the pediatric population. The outcomes reported after a 3 years experience confirm that PEPSiT is a safe, effective, and real minimally invasive procedure to treat adolescents with PSD. It provides patients quick and painless recovery, satisfactory success and high quality of life. A standardized pre- and post-operative protocol, including laser epilation and wound management, is as crucial as the operative technique to achieve successful outcome.
